# Training-Induced Increase in $\overset{\cdot }{V}$O_2max_ and Critical Power, and Acceleration of $\overset{\cdot }{V}$O_2_ on-Kinetics Result from Attenuated P_i_ Increase Caused by Elevated OXPHOS Activity

**DOI:** 10.3390/metabo13111111

**Published:** 2023-10-27

**Authors:** Bernard Korzeniewski

**Affiliations:** BioSimulation Center, PL 30-110 Kraków, Poland; bernard.korzeniewski@gmail.com

**Keywords:** endurance training, inorganic phosphate, $\overset{\cdot }{V}$O_2max_, critical power, $\overset{\cdot }{V}$O_2_ on-kinetics, metabolite homeostasis, computer model

## Abstract

Computer simulations using a dynamic model of the skeletal muscle bioenergetic system, involving the P_i_-double-threshold mechanism of muscle fatigue, demonstrate that the training-induced increase in $\overset{\cdot }{V}$O_2max_, increase in critical power (CP) and acceleration of primary phase II of the $\overset{\cdot }{V}$O_2_ on kinetics (decrease in t_0.63_) is caused by elevated OXPHOS activity acting through a decrease in and slowing of the P_i_ (inorganic phosphate) rise during the rest-to-work transition. This change leads to attenuation of the reaching by P_i_ of Pi_peak_, peak P_i_ at which exercise is terminated because of fatigue. The delayed (in time and in relation to $\overset{\cdot }{V}$O_2_ increase) P_i_ rise for a given power output (PO) in trained muscle causes P_i_ to reach Pi_peak_ (in very heavy exercise) after a longer time and at a higher $\overset{\cdot }{V}$O_2_; thus, exercise duration is lengthened, and $\overset{\cdot }{V}$O_2max_ is elevated compared to untrained muscle. The diminished P_i_ increase during exercise with a given PO can cause P_i_ to stabilize at a steady state less than P_ipeak_, and exercise can continue potentially ad infinitum (heavy exercise), instead of rising unceasingly and ultimately reaching Pi_peak_ and causing exercise termination (very heavy exercise). This outcome means that CP rises, as the given PO is now less than, and not greater than CP. Finally, the diminished P_i_ increase (and other metabolite changes) results in, at a given PO (moderate exercise), the steady state of fluxes (including $\overset{\cdot }{V}$O_2_) and metabolites being reached faster; thus, t_0.63_ is shortened. This effect of elevated OXPHOS activity is possibly somewhat diminished by the training-induced decrease in Pi_peak_.

## 1. Introduction

The maximal oxygen consumption ($\overset{\cdot }{V}$O_2max_), critical power (CP) and $\overset{\cdot }{V}$O_2_ on-kinetics are key properties of skeletal muscle and the whole-body bioenergetic system in humans. $\overset{\cdot }{V}$O_2max_ determines the maximal capacity of oxidative phosphorylation (OXPHOS) for the ATP supply under given conditions; CP corresponds to the maximal work intensity at which a steady state of fluxes (including $\overset{\cdot }{V}$O_2_) and metabolites can be achieved and above which short-term fatigue is initiated; the characteristic transition time t_0.63_ of the primary phase II of the $\overset{\cdot }{V}$O_2_ on-kinetics (time to reach 63% of the $\overset{\cdot }{V}$O_2_ amplitude) describes how fast the system responds to the rest-to-work transition; and the slow component of the $\overset{\cdot }{V}$O_2_ on-kinetics corresponds to an increasing inefficiency of the system related to muscle fatigue that ultimately leads to the termination of exercise. $\overset{\cdot }{V}$O_2max_ and CP are higher, t_0.63_ is shorter, and the slow component is lower in physically active/trained individuals than in sedentary/untrained individuals and especially in older individuals and patients with mitochondrial myopathies and cardiovascular diseases. Therefore, these properties constitute a good and convenient measure of the efficiency of the human bioenergetic system.

Numerous experimental studies have demonstrated that endurance training affects several system properties at the physiological level, for example, increasing the maximal oxygen consumption rate $\overset{\cdot }{V}$O_2max_ [1,2,3,4,5,6,7,8,9,10,11,12,13,14] and augmenting critical power CP [3,4,5], and accelerating primary phase II of $\overset{\cdot }{V}$O_2_ on-kinetics (shortening the transition time t_0.63_) [7,11,13,14,15]. Endurance training also diminishes the $\overset{\cdot }{V}$O_2_ slow component [2,9,11,16].

On the other hand, endurance training also enhances the bioenergetic system at the skeletal muscle cell level, namely elevating the amount/activity of OXPHOS enzymes and whole mitochondria. In particular, training increases total mitochondrial protein [17,18], elevates the amount of enzymes involved in mitochondrial bioenergetics [17,18,19,20], increases the skeletal muscle oxidative/respiratory capacity [19,20,21], augments the OXPHOS activity in mitochondria [10,22], increases the activity of mitochondrial enzymes (complex II, complex III, cytochrome oxidase (COX, complex IV), complex V (ATP synthase), citrate synthase (CS) and ATP/ADP carrier (ANT)) [10,13,14,22,23,24] and increases the mitochondrial volume density [1,8,13,14,23,24,25]. In some cases, the last property is not affected [24].

It was also shown that end-exercise P_i_ at work termination because of fatigue is more than twice lower in trained, compared to untrained, rowers [26], while end-exercise P_i_, H^+^ and H_2_PO_4_^-^ in exhausting exercise are lower in younger individuals than in older people, who can be regarded in a sense as “detrained” individuals [27].

The “P_i_ double-threshold” mechanism of muscle fatigue was postulated recently [28,29]. This mechanism assumes that: (1) the additional ATP usage, underlying the slow component of the $\overset{\cdot }{V}$O_2_ and metabolites on-kinetics, begins when P_i_ exceeds a critical value, Pi_crit_ [28]; (2) muscle work is terminated because of fatigue when P_i_ reaches a peak value, Pi_peak_ [30]; and (3) the increases in P_i_ and additional ATP usage reciprocally stimulate each other, creating a positive feedback loop (self-driving mechanism) [28]. In sufficiently intense exercise, P_i_ ultimately reaches Pi_peak_ (and $\overset{\cdot }{V}$O_2_ reaches $\overset{\cdot }{V}$O_2max_), and exercise is terminated because of exhaustion. The first threshold, corresponding to Pi_crit_ (point 1), the second threshold, corresponding to Pi_peak_ (point 2), and positive feedback (point 3) were introduced previously in relation to an abstract fatigue factor F, representing various fatigue-related metabolites: H^+^, NH_4_^+^, IMP, AMP, ADP, P_i_, etc. [31].

The “P_i_ double-threshold” mechanism is able to generate numerous different, apparently unrelated properties of the skeletal muscle bioenergetic system: time courses of relevant variables, including of muscle (and pulmonary) $\overset{\cdot }{V}$O_2_, cytosolic ADP, pH, PCr and P_i_ during the rest–work transition; the constancy of these variable values at the end of exercise at various power outputs above critical power, the hyperbolic power–duration curve with an asymptote in the form of critical power and the decrease or increase in CP and $\overset{\cdot }{V}$O_2max_ and increase or decrease in t_0.63_ caused by hypoxia or hyperoxia, respectively [28].

The discussed mechanism also allows for consideration of the effect of mutations in mitochondrial and nuclear DNA, leading to impairment of OXPHOS in mitochondrial myopathy (MM) patients regarding the skeletal muscle bioenergetic system and exercise tolerance [32].

The discussed mechanism can also explain the changes in $\overset{\cdot }{V}$O_2max_, CP and $\overset{\cdot }{V}$O_2_ on-kinetics (decrease in t_0.63_ and the slow component) induced by endurance training in healthy persons [29] and MM patients [33]. Computer simulations have predicted that these effects are caused by the training-induced increase in OXPHOS activity. When it is assumed that the increase in OXPHOS activity in vivo corresponds quantitatively to the increase in mitochondria volume density and/or OXPHOS (enzymes) activity in vitro, slightly too great quantitative effects on $\overset{\cdot }{V}$O_2max_ and CP were predicted [29]. Therefore, the possibility was postulated that training also leads to a decrease in Pi_peak_, which diminishes the effect of the increase in OXPHOS activity and improves the metabolite (ADP, P_i_, PCr, H^+^) homeostasis [29,33]. However, alternatively, the increase in the activity of OXPHOS (complexes) measured in a given muscle (e.g., gastrocnemius or quadriceps) in vitro may not be representative of the rise in the (mean) OXPHOS activity in the whole working muscle group (including gluteus, biceps femoris, quadriceps, gastrocnemius and soleus) in vivo [34]. If a smaller training-induced increase in OXPHOS activity in power-generating muscles in vivo is assumed, the training-induced increases in $\overset{\cdot }{V}$O_2max_ and CP encountered in experimental studies can be accounted for quantitatively without the need to decrease Pi_peak_. This problem will have to be resolved by future experimental studies, in particular directed toward the measurement of the effect of training on the end-exercise concentrations of P_i_ and other metabolites (particularly H_2_PO_4_^−^ and H^+^).

The present study is intended to demonstrate how (by which mechanism) the training-induced increase in OXPHOS activity and likely the decrease in Pi_peak_ determine the rises in $\overset{\cdot }{V}$O_2max_ and CP and fall in t_0.63_. It is hypothesized that these changes occur through a delay and decrease in the P_i_ increase during the rest-to-work transition that leads to attenuation of the reaching of Pi_peak_ by P_i_ (the effect of elevated OXPHOS activity on $\overset{\cdot }{V}$O_2max_ and CP) and faster reaching of a new steady state (the effect of elevated OXPHOS activity on t_0.63_) through an accelerated reaching of Pi_peak_ by P_i_ (the effect of lowered Pi_peak_ on $\overset{\cdot }{V}$O_2max_ and CP). It is clearly demonstrated and explicated exactly how this mechanism works.

## 2. Theoretical Methods

### 2.1. Ethical Approval

This study was purely theoretical and did not involve any experiments on humans or animals.

### 2.2. Computer Model

The dynamic computer model of the skeletal muscle bioenergetic system developed previously was used in the present study [28,34,35,36,37,38]. The model involves the each-step activation (ESA) (parallel activation) mechanism of the stimulation of different elements of the bioenergetic system in the cell during work transitions. According to this mechanism, all OXPHOS complexes, NADH supply and glycolysis/glycogenolysis are directly activated by some cytosolic factor/mechanism (which probably involves cytosolic Ca^2+^ ions and possibly protein phosphorylation/dephosphorylation) in parallel with ATP usage activation by Ca^2+^ ions during rest-to-work or low-to-high-work transitions in skeletal muscle, heart and other tissues [39,40,41,42]. Fell and Thomas postulated a similar mechanism, called “multi-site stimulation”, for the regulation of glycolysis and TCA (tricarboxylic acid) cycles [43,44]. The complete model description was published previously [30] and is available on the author’s personal website: http://bernardkorzeniewski.pl (accessed on 22 September 2023).

**Figure 1:**
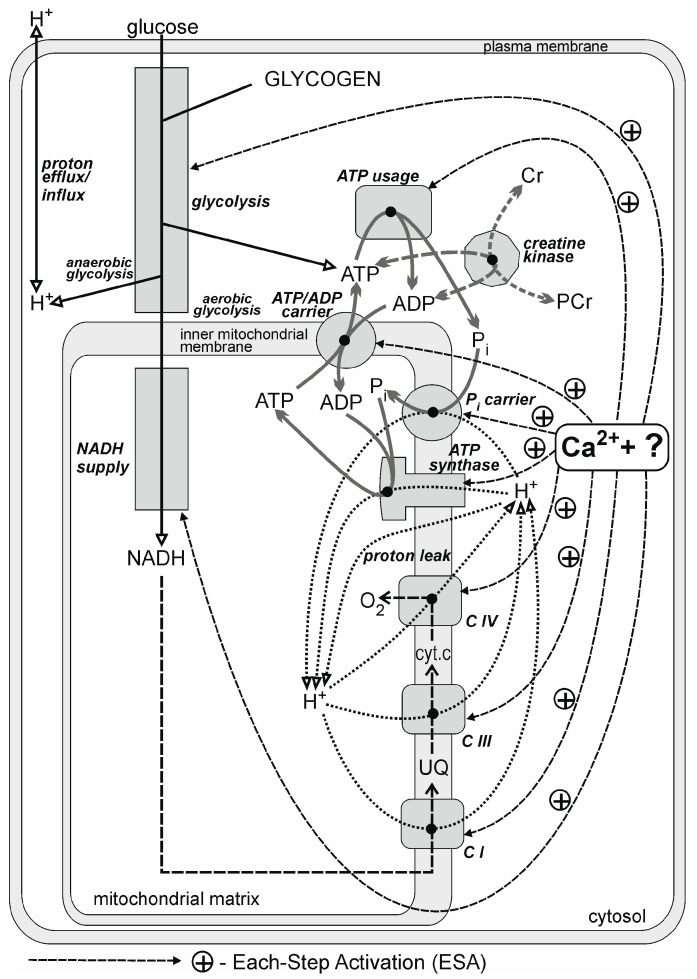
General scheme of the myocyte bioenergetic system. The components of the system are presented that are considered explicitly in the dynamic computer model used for theoretical studies. Each-step activation (ESA) denotes direct activation of (almost) all elements of the system by some mechanism involving cytosolic Ca^2+^ (ATP usage, OXPHOS complexes, malate–aspartate shuttle, MAS and glycolysis) and mitochondrial Ca^2+^ (NADH supply system). Some still unknown factor/mechanism cooperating with Ca^2+^, for example, calmodulin-like protein, which “presents” Ca^2+^ ions to enzymes/carriers and/or protein (de)phosphorylation, is indicated by the question mark (“?”). CI, CIII and CIV indicate complexes I, III and IV of the respiratory chain, respectively; cyt.c, cytochrome c; UQ, ubiquinone. This diagram is taken from [45] (no permission required by the publisher).

A general, simplified scheme of the bioenergetic system in skeletal muscle addressed in the present study is shown in Figure 1. The components of the system that appear explicitly within the model are shown. The two main parts of the model are the set of kinetic equations describing the dependence of the rate of particular enzymatic reactions and processes on metabolite concentrations and the set of ordinary differential equations describing the dependence of the rates of the changes in particular metabolite concentrations on the rates of reactions and processes. In each simulation step (very short time interval), new reaction rates are calculated on the basis of current metabolite concentrations, and new metabolite concentrations are calculated on the basis of current reaction rates.

The action of Ca^2+^ ions and the still unknown additional factor “?” in the system is not involved explicitly in the model but is expressed implicitly as the activity of the regular ATP usage (A_UT_) and activation by ESA of OXPHOS (A_OX_) and glycolysis (A_GL_) (see below). 

This model is able to generate a wide range of various kinetic properties and explain many aspects of the functioning of the skeletal muscle bioenergetic system (see [42] for a review and [28,29,30,32,33,46]).

### 2.3. Bioenergetic Molecular Sequence of Events during Rest-to-Work Transition

The model is intended to reproduce the real behavior of the elements of the system presented in Figure 1. During the rest-to-work transition, the subsequent chain (sequence) of biochemical–molecular events in the skeletal muscle cell bioenergetic system is initiated. Neural myocyte stimulation by an appropriate motor unit leads to a release of Ca^2+^ ions from sarcoplasmic reticulum cisterns. Calcium ions activate actomyosin-ATPase (muscle contraction) and Ca^2+^-ATPase (SERCA; taking up of Ca^2+^ ions during muscle relaxation). As a result, intense hydrolysis of ATP to ADP and P_i_ takes place, and the concentrations of ADP and P_i_ increase. The level of ATP remains almost constant, as the resting ATP/ADP ratio is very high (several hundreds), unless the total adenine nucleotide pool is reduced by AMP deamination. Simultaneously, most cytosolic and mitochondrial elements of the system are directly stimulated by some still unknown factor/mechanism, which probably involves (mostly cytosolic) Ca^2+^ and possibly calmodulin-like proteins presenting Ca^2+^ ions to different enzymes and carriers and/or phosphorylation or dephosphorylation of proteins. The direct stimulation of the ATP supply by ESA attenuates the increases in ADP and P_i_ [40]. Because OXPHOS (together with glycolysis and substrate dehydrogenation) is significantly activated by this mechanism, less accumulation of ADP and P_i_ is needed to stimulate the oxidative and glycolytic ATP supply to match the greatly increased ATP usage for muscle contraction. The equilibrium of the very fast reaction catalyzed by creatine kinase (CK) is shifted as a result of the moderate ADP increase. Consequently, a moderate fall in PCr, rise in Cr, consumption of protons (transient initial pH increase) and further moderate rise in P_i_ (resulting from the co-operation of creatine kinase and ATP usage) take place. The rises in ADP and P_i_ further drive OXPHOS, resulting in augmentation of $\overset{\cdot }{V}$O_2_, which is simultaneously stimulated through the direct OXPHOS activation by ESA. As the changes in metabolite concentrations, especially PCr, Cr and P_i_, are only moderate, the characteristic transition time of primary phase II of the $\overset{\cdot }{V}$O_2_ (and metabolites) on-kinetics (t_0.63_) is rather short. The increases in ADP and AMP (and other metabolites not considered explicitly within the model) additionally stimulate (anaerobic) glycolysis. The production of H^+^ ions by anaerobic glycolysis leads to a decrease in pH to less than its resting value. The magnitude of this acidification depends on exercise intensity: the greater that the power output is, the stronger that the acidification is. However, accumulating H^+^ ions inhibit (anaerobic) glycolysis, preventing further significant cytosol acidification (self-limiting process). In the moderate exercise intensity domain, the system ultimately reaches a steady state (see [45] for more details).

In heavy, very heavy and severe exercise intensity domains [47] additional biochemical–molecular events in the muscle bioenergetic system form a causal chain (sequence) supplementing the processes occurring in the primary phase II on-kinetics of the system. In particular, the slow component of the $\overset{\cdot }{V}$O_2_ and metabolite on-kinetics appears. A sufficiently high work intensity (ATP usage activity) causes P_i_ to exceed Pi_crit_, which starts the additional ATP usage (as opposed to the regular ATP usage related to power generation). This increase is associated with additional rises in ADP and P_i_, and P_i_ further enhances the additional ATP usage, leading to the formation of a positive feedback loop (self-driving phenomenon). The additional increase in ADP further affects the creatine kinase equilibrium and results in an additional fall in PCr and rise in Cr. The further elevated ADP and AMP additionally enhance anaerobic glycolysis, leading to greater cytosolic acidification. Accumulating protons in turn recursively inhibit (anaerobic) glycolysis. ADP and P_i_ rise continuously, further stimulating OXPHOS and leading to an additional increase in $\overset{\cdot }{V}$O_2_. Consequently, the slow component of the on-kinetics of oxygen consumption and metabolites appears. The mutual stimulation of the rise in P_i_ and additional ATP usage in heavy exercise are not intense enough to reach Pi_peak_ by P_i_ and to reach of $\overset{\cdot }{V}$O_2max_ by $\overset{\cdot }{V}$O_2_. Consequently, exercise is not terminated because of fatigue, and the system finally approaches a steady state, although a higher one than that achieved without the presence of the additional ATP usage and $\overset{\cdot }{V}$O_2_ and metabolites’ slow components. The heavy/very heavy exercise border constitutes an emerging property of the system that separates work intensities/A_UT_s, for which this feedback loop leads to P_i_ stabilization at less than Pi_peak_ from those for which it does not. This border represents the critical ATP usage activity (A_UTcrit_) at the muscle level and critical power (CP) at the whole-body level. With very heavy and severe exercise, the reciprocal driving of the rises in P_i_ and additional ATP usage is intense enough to prevent reaching of a steady state. Metabolites change, and oxygen consumption increases continuously. Finally, when P_i_ reaches Pi_peak_, and $\overset{\cdot }{V}$O_2_ reaches $\overset{\cdot }{V}$O_2max_, muscle work is terminated because of exhaustion (see [45] for more details).

It should be emphasized that the $\overset{\cdot }{V}$O_2_ kinetics constitute a result of this sequence of events; therefore, it cannot be a cause of any system property. On the contrary, it is an epiphenomenon (emergent property of the system) that can serve as a non-invasive indicator of the biochemical/kinetic properties/events originating in the muscle, especially of total OXPHOS activity ([45,46]; see below).

### 2.4. Computer Simulations

The ATP usage activity (A_UT_, proportional to power output) is scaled to 1 at rest. This regular ATP usage differs from the additional ATP usage, which underlies the slow component of $\overset{\cdot }{V}$O_2_ and metabolites. At the onset of constant-power exercise, it is elevated instantaneously to a determined value, for example, 100 for intense exercise. One A_UT_ unit is an equivalent of roughly 3 W (2–4 W depending, for instance, on working muscle mass) in whole-body exercise, such as cycling or running. 

Rate constants for OXPHOS complexes and NADH supply block present in kinetic equations in the computer model can be represented as a single rate constant of OXPHOS (k_OX_), representing the OXPHOS activity. This rate constant is scaled to 1 in the “standard” version of the model for young, physically active individuals. At the onset of exercise, this “default” OXPHOS activity (at rest and during work) is multiplied by the ESA intensity A_OX_, being a saturating function of ATP usage activity A_UT_ [37,38]. This activity can be called the “work-induced” OXPHOS activity (present only during work). Therefore, the total OXPHOS activity = default OXPHOS activity × induced OXPHOS activity (ESA intensity). During work, OXPHOS is additionally moderately stimulated by the ADP and P_i_ increases. A_OX_ is elevated through an increase in the parameter A_OXmax_, which can be called the ESA rate constant, at the onset of exercise. OXPHOS complexes, NADH supply block and glycolysis are activated by ESA with some delay in parallel with ATP usage, the activity of which is elevated step-wise (see, e.g., [36,39,40,41,42]).

Within the model, the “P_i_ double-threshold” mechanism of muscle fatigue is expressed by a fixed Pi_crit_ = 18 mM, Pi_peak_ = 25 mM (in the “standard” version of the model for young physically-active individuals) and kinetic equation for the additional ATP usage (for P_i_ > Pi_crit_), in which the additional ATP usage flux is proportional to the current P_i_−Pi_crit_ difference [28,47]. The kinetic equation for the intensity of the additional ATP usage has the following form:

(1) $$v_{add}=k_{add}\cdot v_{UT}\cdot {\left(P_{i}-P_{i_{crit}}\right)}^{0.5}\cdot e^{-t_{a}/t_{add}}$$

where v_add_ is the rate of additional ATP usage (mM min^−1^), k_add_ = 0.2 mM^−0.5^ is the activity (“rate constant”) of the additional ATP usage, v_UT_ is the rate of the regular (as opposed to additional) ATP usage (mM min^−1^), P_i_ is the current inorganic phosphate concentration (mM), t_a_ = 2 min is the characteristic time of the activation of the additional ATP usage, and t_add_ is the time after the onset of exercise.

The computer model involves a constant capillary O_2_ concentration during exercise equal to 30 μM in the standard model version.

In the present study, the following simulations demonstrating the effect of endurance training on the key variables of the skeletal muscle bioenergetic system were performed. 

In the simulations of the effect of training on $\overset{\cdot }{V}$O_2max_, the activity of ATP usage (work intensity) A_UT_ = 90 was used, representing the very heavy exercise-intensity domain both before and after training. The “default” activity of OXPHOS was augmented by 10% (k_OX_: 1.0 → 1.1), while ESA intensity (“work-induced” OXPHOS activity) was assumed to be unchanged. The “standard” value of Pi_peak_ = 25 mM was used.

In the simulations of the effect of training on the critical ATP usage activity (A_UTcrit_, analogous to CP) the activity of ATP usage (work intensity) A_UT_ = 82 was used, which represented the very heavy exercise-intensity domain before training and heavy exercise-intensity domain after training. The “default” activity of OXPHOS was augmented by 10% (k_OX_: 1.0 → 1.1), while ESA intensity (“work-induced” OXPHOS activity) was assumed to be unchanged. The “standard” value of Pi_peak_ = 25 mM was used.

In the simulations of the effect of training on t_0.63_, the activity of ATP usage (work intensity) A_UT_ = 50 was used, representing the moderate exercise-intensity domain both before and after training. The “default” activity of OXPHOS was augmented by 22% (k_OX_: 0.9 → 1.1), while ESA intensity (“work-induced” OXPHOS activity) was assumed to be unchanged. The “standard” value of Pi_peak_ = 25 mM was used.

In the simulations of the effect of the Pi_peak_ decrease in trained muscle on $\overset{\cdot }{V}$O_2max_, the activity of ATP usage (work intensity) A_UT_ = 90 was used, representing the very heavy exercise-intensity domain. The “trained” OXPHOS activity was used (k_OX_ = 1.1), and Pi_peak_ was decreased: 25 mM → 22.5 mM.

In the simulations of the effect of the Pi_peak_ decrease in trained muscle on CP, the activity of ATP usage (work intensity) A_UT_ = 82 was used, representing the heavy exercise-intensity domain without Pi_peak_ decrease and very heavy exercise-intensity domain with Pi_peak_ decrease. The “trained” OXPHOS activity was used (k_OX_ = 1.1), and Pi_peak_ was decreased: 25 mM → 22 mM.

The values of the parameters A_UT_, k_OX_ and Pi_peak_ and their changes were chosen arbitrarily to enable a clear presentation.

## 3. Results

**Figure 2:**
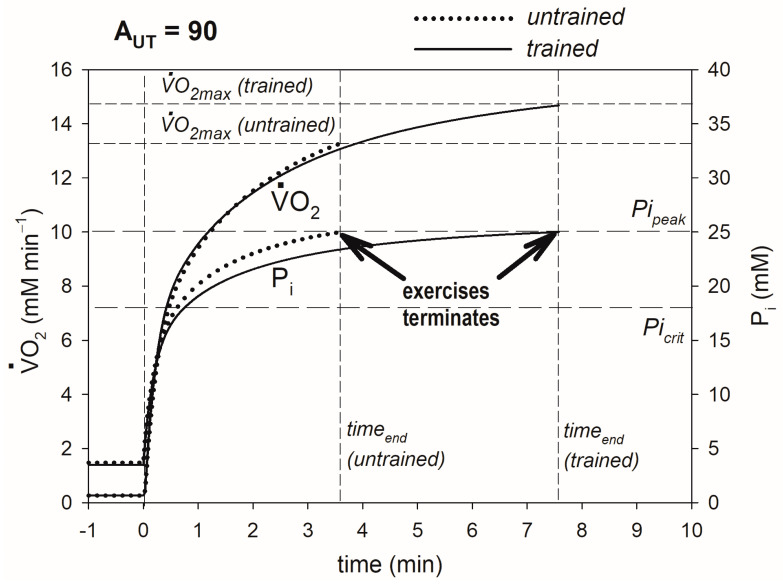
Simulated effect of training-induced increase in OXPHOS activity on $\overset{\cdot }{V}$O_2max_. Time courses of muscle $\overset{\cdot }{V}$O_2_ and P_i_ in untrained and trained muscle are shown. $\overset{\cdot }{V}$O_2max_ and end-exercise time before and after training are indicated. The system remains in the very heavy exercise-intensity domain (A_UT_ = 90 is greater than A_UTcrit_ both before and after training). The additional ATP usage, underlying the slow component of the $\overset{\cdot }{V}$O_2_ and P_i_ slow component, is launched when P_i_ exceeds Pi_crit_, and exercise is terminated because of fatigue when P_i_ reaches Pi_peak_.

Muscle training, leading to an increase in OXPHOS activity, elevates $\overset{\cdot }{V}$O_2max_ and lengthens the duration of exercise in very heavy exercise. This outcome is demonstrated if Figure 2. It occurs through attenuation (delay and decrease in relation of $\overset{\cdot }{V}$O_2_) of the P_i_ rise during exercise. As a result, in trained muscle, P_i_ reaches Pi_peak_, and exercise is terminated because of fatigue after a longer time and at a higher $\overset{\cdot }{V}$O_2_ ($\overset{\cdot }{V}$O_2max_) than in untrained muscle. Thus, at a given work intensity (regular ATP usage activity), $\overset{\cdot }{V}$O_2max_ is augmented, and the duration of exercise is lengthened.

**Figure 3:**
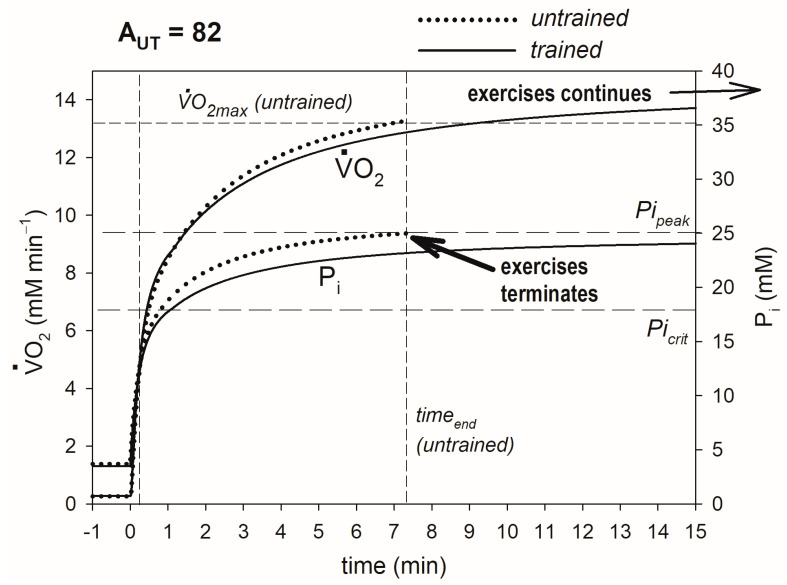
Simulated effect of training-induced increase in OXPHOS activity on CP (A_UTcrit_). Time courses of muscle $\overset{\cdot }{V}$O_2_ and P_i_ in untrained and trained muscle are shown. $\overset{\cdot }{V}$O_2max_ and end-exercise time before training are indicated. After training, P_i_ reaches a steady state less than Pi_peak_, $\overset{\cdot }{V}$O_2_ reaches a steady state less than $\overset{\cdot }{V}$O_2max_, and exercise is not terminated because of fatigue: the system passes from the very heavy to heavy exercise intensity domain (A_UT_ = 82 is greater than A_UTcrit_ before training and less than A_UTcrit_ after training). The additional ATP usage, underlying the slow component of the $\overset{\cdot }{V}$O_2_ and P_i_ slow component, is initiated when P_i_ exceeds Pi_crit_, and exercise is terminated because of fatigue when P_i_ reaches Pi_peak_.

The training-induced increase in OXPHOS activity also elevates A_UTcrit_ (critical ATP usage activity proportional to critical power, CP) and can lead to the transition of exercise of a given intensity (power output) from the very heavy-intensity domain to heavy-intensity domain. This outcome is demonstrated if Figure 3. It occurs because of a decrease in the P_i_ rise during exercise. Before training, P_i_ was unceasingly rising and ultimately reached P_i_ and thus caused exercise termination because of fatigue at this power output (PO), while after training, P_i_ was increasing at a slower pace and finally stabilized at a steady-state value less than Pi_peak_, and exercise could be continued potentially ad infinitum. As a result, in trained muscle, $\overset{\cdot }{V}$O_2_ does not reach $\overset{\cdot }{V}$O_2max_ and stabilizes at a steady-state value less than it, in opposition to the situation taking place in untrained muscle. Thus, as the result of training, CP is elevated, and PO, which was greater than CP in untrained muscle, can be less than CP in trained muscle.

**Figure 4:**
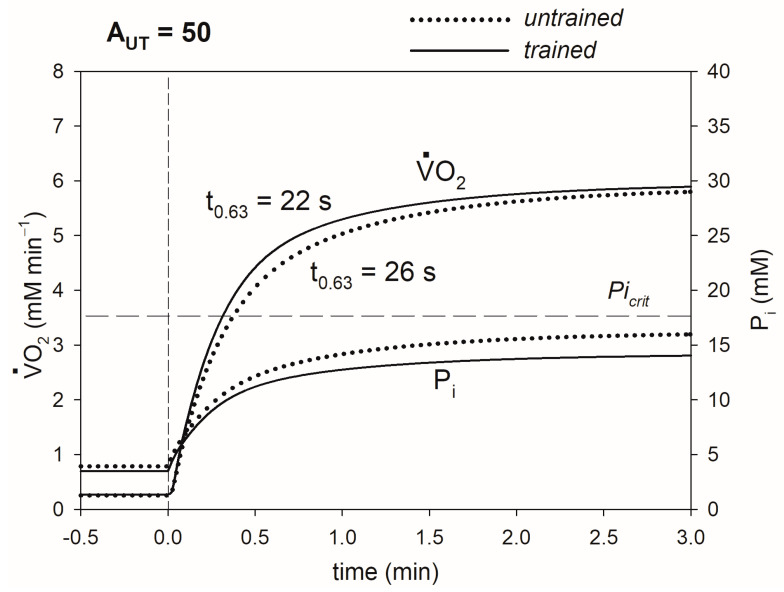
Simulated effect of training-induced increase in OXPHOS activity on t_0.63_. Time courses of muscle $\overset{\cdot }{V}$O_2_ and P_i_ in untrained and trained muscle are shown. t_0.63_ before and after training is shown. The system remains in the moderate exercise intensity domain (A_UT_ = 50 is less than A_UTadd_, A_UT_ at which P_i_ exceeds Pi_crit_, and the additional ATP usage, underlying the slow component of the $\overset{\cdot }{V}$O_2_ and P_i_ slow component, is launched, both before and after training).

The elevated OXPHOS activity as the result of training shortens the transition time of primary phase II of the $\overset{\cdot }{V}$O_2_ on-kinetics t_0.63_, which is best seen in the moderate exercise-intensity domain, where the $\overset{\cdot }{V}$O_2_ slow component and the additional ATP usage underlying it are absent. This outcome is demonstrated in Figure 4. This effect is mostly caused by the decrease in the P_i_ increase (associated with a smaller PCr decrease/Cr increase) (e.g., the smaller training-induced increase in ADP plays a minor role, as the ADP concentration is in the micromolar range; see Discussion). Here, both before and after training, exercise is within the moderate-intensity domain. However, for some power outputs, muscle training can bring the system from the (very) heavy exercise-intensity domain to the moderate exercise-intensity domain (see [29], Figure 6 therein).

**Figure 5:**
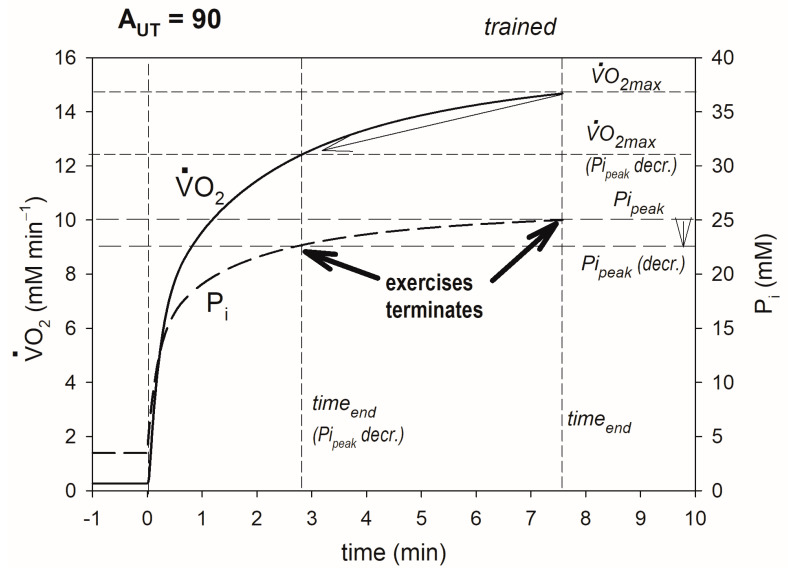
Simulated effect of training-induced decrease in Pi_peak_ (Pi_peak_ decr.) on $\overset{\cdot }{V}$O_2max_. OXPHOS activity is increased as the result of training, as in Figure 2. Time courses of muscle $\overset{\cdot }{V}$O_2_ and P_i_ in trained muscle without and with Pi_peak_ decreases are shown. The lines representing $\overset{\cdot }{V}$O_2_ and P_i_ overlap at the moment when exercise is terminated at lowered Pi_peak_. $\overset{\cdot }{V}$O_2max_ and time_end_ are decreased as a result of the Pi_peak_ decrease. The additional ATP usage, underlying the slow component of $\overset{\cdot }{V}$O_2_ and the P_i_ slow component, is initiated when P_i_ exceeds Pi_crit_, and exercise is terminated because of fatigue when P_i_ reaches Pi_peak_.

The possible training-induced decrease in Pi_peak_ weakens the rise in $\overset{\cdot }{V}$O_2_ caused by the OXPHOS activity increase. This outcome is demonstrated in Figure 5. In the presence of the diminished Pi_peak_, P_i_ simply reaches Pi_peak_, and exercise is terminated because of fatigue earlier after the onset of exercise and at a lower $\overset{\cdot }{V}$O_2_. Therefore, time_end_ and $\overset{\cdot }{V}$O_2max_ decrease. For this reason, generally, the training-induced increases in $\overset{\cdot }{V}$O_2max_ and time_end_ are smaller than they would be if only OXPHOS activity increased, and Pi_peak_ did not decrease. Nevertheless, exercise remains in the very heavy-intensity domain.

**Figure 6:**
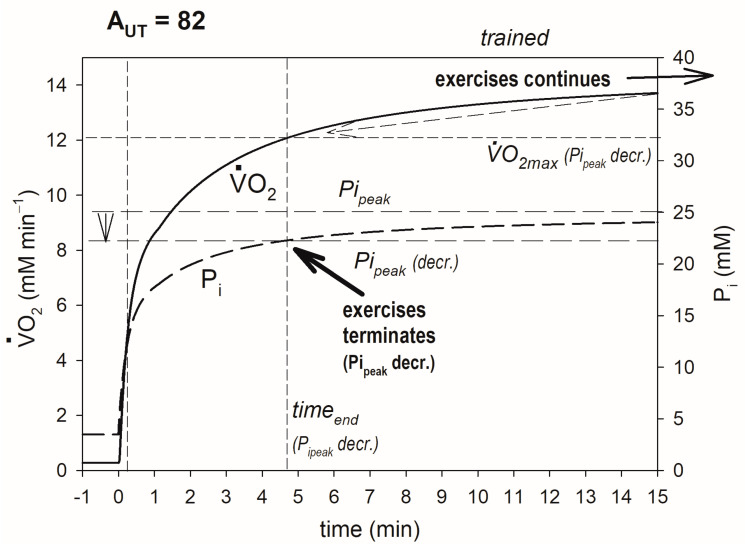
Simulated effect of training-induced decrease in Pi_peak_ (Pi_peak_ decr.) on CP (A_UTcrit_). OXPHOS activity is increased as the result of training, as in Figure 3. Time courses of muscle $\overset{\cdot }{V}$O_2_ and P_i_ in trained muscle without and with Pi_peak_ decreases are shown. The lines representing $\overset{\cdot }{V}$O_2_ and P_i_ overlap at the moment when exercise is terminated at lowered Pi_peak_. The Pi_peak_ decrease lowers A_UTcrit_, causing A_UT_ = 82 to be greater than A_UTcrit_, P_i_ reaches (decreased) Pi_peak_, $\overset{\cdot }{V}$O_2_ reaches (decreased) $\overset{\cdot }{V}$O_2max_ and the system passes from the heavy to very heavy exercise-intensity domain. Exercise is terminated because of fatigue when P_i_ reaches (lowered) Pi_peak_.

The possible training-induced decrease in Pi_peak_ diminishes the increase in CP caused by the OXPHOS activity increase. This outcome is demonstrated in Figure 6. In the presence of the diminished Pi_peak_ at a given power output, P_i_ does not stabilize at a steady state less than Pi_peak_ but reaches Pi_peak_, and exercise does not continue potentially ad infinitum but is terminated because of fatigue at time_end_. PO, which was less than CP in the absence of the Pi_peak_ decrease, is now greater than (diminished) CP when Pi_peak_ falls. Therefore, generally, the training-induced increase in CP is smaller than it would be if only OXPHOS activity increased, and Pi_peak_ did not decrease. Exercise of a given intensity passes from the heavy-intensity domain to the very heavy-intensity domain.

The decrease in Pi_peak_ does not affect t_0.63_, as the primary phase II of $\overset{\cdot }{V}$O_2_ on-kinetics does not depend on Pi_peak_.

## 4. Discussion

### 4.1. Mechanism of the Impact of Training-Induced Increase in OXPHOS Activity and Decrease in Pi_peak_ on $\overset{\cdot }{V}$O_2max_, CP and t_0.63_

The aim of the present article is to explain and explicate in detail the mechanisms through which the training-induced increase in OXPHOS activity and likely decrease in Pi_peak_ determine the increases in $\overset{\cdot }{V}$O_2max_ and CP and decrease in t_0.63_. Previous in silico studies [29,33,45] demonstrated that the rise in OXPHOS activity led to rises in $\overset{\cdot }{V}$O_2max_ and CP and a fall in t_0.63_, while the decrease in Pi_peak_ diminishes the increases in $\overset{\cdot }{V}$O_2max_ and CP. However, the detailed mechanism underlying this effect was not explicated. Additionally, previous studies did not address the effect of the OXPHOS activity and peak P_i_ on exercise duration until exhaustion.

The present article demonstrates that the effect of the training-induced increase in OXPHOS activity on $\overset{\cdot }{V}$O_2max_, CP and t_0.63_ and of the decrease in Pi_peak_ on $\overset{\cdot }{V}$O_2max_ and CP is mediated through the attenuation (decrease and delay) of the P_i_ increase after the onset of exercise. This attenuation is caused by the increase in OXPHOS activity and is possibly partly compensated for (diminished) by the Pi_peak_ decrease. 

**Figure 7:**
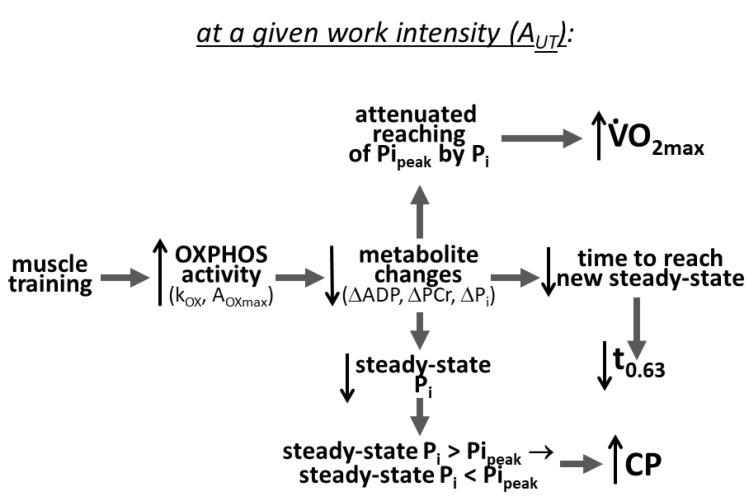
Mechanism of the training-induced increases in $\overset{\cdot }{V}$O_2max_, CP and t_0.63_. Muscle training causes an increase in total (default and/or work-induced) OXPHOS activity, leading to smaller changes in metabolites (ADP, P_i_, PCr, H^+^) at a given time after the onset of exercise (delay/attenuation of metabolite changes). As a result: 1. The increase in P_i_ with time (especially in relation to the increase in $\overset{\cdot }{V}$O_2_) and reaching of Pi_peak_ by P_i_ are attenuated; $\overset{\cdot }{V}$O_2_ can increase more at a given P_i_, and thus, $\overset{\cdot }{V}$O_2_ at Pi_peak_, that is $\overset{\cdot }{V}$O_2max_, is elevated; 2. At a given work intensity (ATP usage activity, A_UT_), the new steady state of primary phase II of metabolites (ADP, P_i_, PCr, H^+^) on-kinetics is reached in a shorter time, OXPHOS is stimulated faster by increases in ADP and P_i_, and therefore, the transition time t_0.63_ of the $\overset{\cdot }{V}$O_2_ on-kinetics is shortened; 3. The steady state of P_i_ concentration (not reached for PO greater than CP, that is, for A_UT_ greater than A_UTcrit_) can fall, for a given A_UT_, from greater than to less than Pi_peak_ so that the system passes from the very heavy/severe to heavy, or even moderate exercise intensity domain, and thus, A_UTcrit_ (CP) is elevated.

The details of the mechanism of the impact of the elevated OXPHOS activity on $\overset{\cdot }{V}$O_2max_, CP and t_0.63_ are presented in Figure 2, Figure 3 and Figure 4 and are summarized in Figure 7. 

The training-induced increase in the total OXPHOS activity can occur through an increase in the “default” OXPHOS activity k_OX_ (see Introduction), an increase in the “work-induced” OXPHOS activity (ESA intensity, A_OX_) [13] or both. The present study focuses on the former, but the general reasoning would be very similar for the latter.

The training-induced rise in OXPHOS activity leads to attenuation (decrease and delay) of changes in the bioenergetic system metabolites (e.g., increases in ADP, P_i_, Cr and H^+^ and decrease in PCr) during the rest-to-work transition at a given power output. In particular, this change concerns the increase in P_i_: it rises at a slower pace and to a smaller extent at a given $\overset{\cdot }{V}$O_2_ (Figure 2). During the rest-to-work transition, OXPHOS with the default (resting) activity (k_OX_) is very quickly (in a few seconds) stimulated by ESA (work-induced OXPHOS activity, A_OX_) and then more slowly (tens of seconds to minutes) by increases in ADP and P_i_. In the result of the training-induced rise in the total OXPHOS activity (k_OX_ and/or A_OX_), P_i_ (and ADP) does not have to increase as much to stimulate OXPHOS (oxidative ATP supply to match the elevated ATP usage), as its activity is already elevated by training. As a result, in trained muscle, P_i_ increases at a slower pace and reaches Pi_peak_ (during very heavy and severe exercise), and exercise is terminated because of fatigue after a longer time and at a higher $\overset{\cdot }{V}$O_2_ than in untrained muscle. Because of the elevated OXPHOS activity, the P_i_ increase is delayed in relation to the $\overset{\cdot }{V}$O_2_ increase, or alternatively, $\overset{\cdot }{V}$O_2_ increases more at a given P_i_ increase until P_i_ reaches Pi_peak_. Consequently, the exercise duration until exhaustion time_end_ is lengthened, and $\overset{\cdot }{V}$O_2max_ is elevated. The system remains in the very heavy exercise-intensity domain.

The training-induced slowed and decreased P_i_ increase during exercise (due to augmented OXPHOS activity) can cause, at a given power output (PO), the steady-state P_i_ value to decrease: P_i_ ultimately stabilizes at a steady-state value less than Pi_peak_, and exercise continues potentially ad infinitum, instead of rising unceasingly until reaching Pi_peak_ and causing exercise termination. This outcome is demonstrated in Figure 3 and Figure 7. In other words, muscle training causes the PO value, which was greater than CP (critical power) before training, to be smaller than CP after training. Consequently, exercise of a given intensity passes from the very heavy to heavy, or even moderate, domain.

The decreased magnitude of the P_i_ increase during exercise of a given intensity also leads to a shortening of the transition time of primary phase II of the $\overset{\cdot }{V}$O_2_ on-kinetics: t_0.63_. Easterby [48] showed in an abstract and general way that the time of the transition between steady states in a metabolic system is proportional to the changes in metabolite concentrations during this transition. It was demonstrated in the concrete case of the skeletal muscle bioenergetic system that t_0.63_ of the $\overset{\cdot }{V}$O_2_ on-kinetics depends near-linearly on the changes in, first of all, PCr, Cr and P_i_ (anyway, related to each other) during the rest-to-work transition at the same PO [49] (ADP and its changes are in the micromolar range and therefore contribute little to the general effect, while ATP is essentially constant in the absence of AMP deamination). At a given metabolic flux, the smaller that the changes in metabolites are, the faster that the new steady-state is reached, and consequently the shorter that the transition time is. The effect of the smaller P_i_ increase on t_0.63_ is demonstrated in Figure 4 (and summarized in Figure 7) regarding the example of moderate exercise, where the additional ATP usage and slow component of the $\overset{\cdot }{V}$O_2_ on-kinetics are absent, facilitating a clear presentation.

The possible training-induced decrease in Pi_peak_ counterbalances to a certain extent the effect of elevated OXPHOS activity. In the presence of a lowered Pi_peak_, P_i_ reaches Pi_peak_ in a shorter time and at a lower $\overset{\cdot }{V}$O_2_, than at unaltered Pi_peak_, leading to the shortening of exercise duration and a fall in $\overset{\cdot }{V}$O_2max_. This outcome is demonstrated in Figure 5. This effect takes place within the very heavy exercise-intensity domain.

At some lower PO values, the decrease in Pi_peak_ can lead to a transition from the heavy to very heavy exercise-intensity domain. The P_i_ concentration, which did not reach (unchanged) Pi_peak_ in the absence of the Pi_peak_ decrease, can be greater than (lowered) Pi_peak_ in the presence of the Pi_peak_ decrease. Therefore, while in the former case, exercise could be continued potentially ad infinitum, in the latter case, it is terminated after some time because of fatigue. This outcome is demonstrated in Figure 6 and is equivalent to a CP decrease. Therefore, a given PO can be less than CP in the absence of the Pi_peak_ fall and greater than CP in the presence of this fall.

As mentioned above, as Pi_peak_ does not affect the reaching of a steady state of P_i_ and $\overset{\cdot }{V}$O_2_ in primary phase II of the $\overset{\cdot }{V}$O_2_ and P_i_ on-kinetics, changes in Pi_peak_ have no effect on t_0.63_. 

Summing up, the training-induced increase in the “default” OXPHOS activity and possibly “work-induced” OXPHOS activity (ESA intensity) acts through a decrease and delay in metabolite (ADP, P_i_, PCr, H^+^, AMP, IMP, NH_3_) changes, especially the P_i_ increase during the rest-to-work transition (improvement in metabolite homeostasis, at least at a given time and $\overset{\cdot }{V}$O_2_). This outcome in turn leads to an increase in $\overset{\cdot }{V}$O_2max_, increase in CP and shortening of t_0.63_ (Figure 7). The effect on $\overset{\cdot }{V}$O_2max_ and CP can be diminished by a training-induced decrease in Pi_peak_, which is associated with improved end-exercise metabolite homeostasis.

### 4.2. General Discussion

The present article addresses the training-induced changes in $\overset{\cdot }{V}$O_2max_, CP (A_UTcrit_) and t_0.63_ at the muscle level. However, the kinetic properties of the system at the whole-body, including the pulmonary, level can to a certain degree differ from their counterparts at the muscle level. First, the pulmonary and muscle $\overset{\cdot }{V}$O_2_ kinetics can somewhat dissociate, for instance, during very intense exercise or off-transient. This outcome can be caused by some delays in oxygen transport by the circulatory system from working muscles to the lungs, buffering of the O_2_ level by oxygen stores in tissues, blood and lungs or contributions of oxygen consumption by auxiliary tissues (heart, respiratory muscle, posture-maintaining muscles) to the pulmonary oxygen consumption [45]. Second, it is possible that the moderate/heavy exercise border at the whole-body level (lactate threshold, LT, ventilatory threshold, VT) appears earlier (in time and at a lower PO) than at the working muscle level (A_UTadd_, ATP usage activity at which the additional ATP usage, underlying the $\overset{\cdot }{V}$O_2_ and metabolites’ slow component, is initiated) [45]. 

Previous in silico studies [29,33] demonstrated that the training-induced increase in OXPHOS activity and/or ESA intensity not only elevates the critical ATP usage activity (A_UTcrit_, analogous to CP) but shifts the whole power–duration dependence upward (toward higher values of A_UTcrit_/PO). At the same time, depending on detailed parameter values (particularly changes in k_OX_ and Pi_peak_), the curvature constant W’ of the power–duration relationship (equivalent to the slope of the linear A_UT_-1/time relationship) remains essentially unaffected by training or somewhat decreases. Both cases were encountered in experimental studies [3,4,5]. Of course, this effect can be explained in terms of the impact on the P_i_ increase kinetics during the rest-to-work transition, which affects both CP and duration of exercise, as demonstrated above.

Only the effect of Pi_peak_ on the training-induced changes in the kinetic properties of the skeletal muscle bioenergetic system elicited through OXPHOS activity enhancement and attenuation of the P_i_ increase during exercise was analyzed in the present study. However, it cannot be excluded that other parameters, for example, Pi_crit_ (critical P_i_, above which the additional ATP usage, underlying the $\overset{\cdot }{V}$O_2_ and metabolites’ slow component, is initiated) or k_add_ (the activity or “rate constant” of the additional ATP usage), are affected by muscle training. It was demonstrated that an increase in Pi_crit_ elevates $\overset{\cdot }{V}$O_2_ and CP and lowers t_0.63_ [29,46]. It also reduces the slow component of $\overset{\cdot }{V}$O_2_ (and metabolites) on-kinetics. A rise in k_add_ diminishes CP and does not affect $\overset{\cdot }{V}$O_2max_ or t_0.63_ [29,46]. Of course, it enlarges the slow component intensity. The mechanism of the potential action of Pi_crit_ and k_add_ will be discussed and explained in detail when evidence for/reasons to believe in training-induced changes in these parameters appear. 

The $\overset{\cdot }{V}$O_2_ on-kinetics (t_0.63_ and/or O_2_ deficit) was proposed recently [50] to determine CP. However, $\overset{\cdot }{V}$O_2_ and its kinetics are emergent properties of the system and can only be correlated with, but they do not bring about (determine) anything within the system. On the contrary, t_0.63_ and CP result from system parameters, such as OXPHOS activity and Pi_peak_, acting through the P_i_ on-kinetics, as demonstrated in the present in silico study. The inverse correlation between t_0.63_ and CP encountered in experimental studies results from, e.g., OXPHOS activity changing t_0.63_ and CP (A_UTcrit_) in the opposite directions [46]. Additionally, it was postulated [50] that t_0.63_ determines metabolite changes during the rest-to-work transition. However, in reality, the relation is just the opposite: this is changes in the metabolites (especially PCr, Cr and P_i_) that determine t_0.63_ at a given work intensity (see above [45,49]). 

Of course, the possibility of a training-induced Pi_peak_ decrease can be tested experimentally, for example, by measuring the end-exercise P_i_ concentration in very heavy exercise before and after training.

### 4.3. Study Limitations

Every computer model of a complex biochemical/cellular/physiological system constitutes at best only a simplification and approximation of the reality. Of course, this fact also concerns the dynamic model of the skeletal muscle bioenergetic system used in the present study. 

The model describes only one compartment corresponding to working muscles and does not distinguish particular working (power-generating) muscles (including the gluteus, quadriceps, biceps femoris, gastrocnemius and soleus, the kinetic/metabolic properties of which can differ to a certain extent) and different muscle fiber types within muscles (type I, IIa and IIx fibers and their various sub-types), and it involves parameters and variables (rate constants, activities, fluxes, metabolite concentrations) that are averaged over the entire working muscles group and particular muscles. Nevertheless, the model is compared with experimental data concerning muscle (or pulmonary) $\overset{\cdot }{V}$O_2_ and muscle PCr, P_i_, ADP, ATP and H^+^ concentrations averaged over the entire muscle. Even then, the model can generate, at least semi-quantitatively, a surprisingly broad set of dynamic properties of the modeled system.

Only the total P_i_ concentration as the main fatigue factor is considered explicitly by the “P_i_ double-threshold” mechanism. It was postulated as a major fatigue-related factor in peripheral fatigue [51]. Nevertheless, other metabolites, such as H^+^, ADP, NH_4_^+^, IMP and AMP, can also contribute to peripheral muscle fatigue [51]. On the other hand, the levels of these metabolites (at least H^+^ and ADP) change together with P_i_ during exercise [28,29]. Therefore, P_i_ can be regarded as a “representative” of various metabolites causing muscle fatigue. Some authors [28,52] have proposed that rather than H_2_PO_4_^−^, a deprotonated form of P_i_, and not P_i_ itself, is the most important fatigue-related factor. H_2_PO_4_^−^ seems to be an attractive candidate, as its relative increase during the rest-to-work transition is greater than that of P_i_ [45], and it represents the increase in both P_i_ and H^+^ (pH decrease increases the fraction of P_i_ in the form of H_2_PO_4_^−^), regarded as the two most important fatigue factors [51]. A substitution within the computer model of P_i_ by H_2_PO_4_^−^ as the fatigue factor provided similar general results. Moreover, altered Ca^2+^ sensitivity was postulated to contribute to peripheral fatigue generation [51,53]. However, P_i_ can cause Ca^2+^ precipitation in sarcoplasmic reticulum [53]. Additionally, one can speculate that P_i_ (and other related metabolites) can be potentially involved in central fatigue, as the central nervous system can detect the metabolic state of working muscle cells, for instance, through type III/IV afferents [30]. Moreover, one could speculate that, for example, mental fatigue, sleepiness or illness can co-determine the Pi_peak_ and/or Pi_crit_ fixed by the brain. Therefore, P_i_ as the main, or at least representative, fatigue-related factor seems to be quite a satisfactory approximation, as it leads to astonishingly good agreement of computer simulations with various experimental data and can account for different, seemingly unrelated, features of the system.

## 5. Conclusions

The training-induced increase in $\overset{\cdot }{V}$O_2max_, increase in critical power (CP) and acceleration of $\overset{\cdot }{V}$O_2_ on-kinetics (decrease in t_0.63_) in the skeletal muscle bioenergetic system caused by a rise in OXPHOS activity are mediated by attenuation (delay and decrease) of the rise in P_i_ (inorganic phosphate) after the onset exercise. This outcome delays the reaching of Pi_peak_ (peak P_i_) by P_i_ and termination of exercise because of fatigue, and it lowers P_i_ at a given $\overset{\cdot }{V}$O_2_, causing a higher $\overset{\cdot }{V}$O_2_ to be reached at the end of exercise, when P_i_ reaches Pi_peak_. This outcome is equivalent to the increase in the duration of exercise of a given power output and elevation of $\overset{\cdot }{V}$O_2max_. Additionally, the decrease/delay in trained muscle of the P_i_ increase during exercise of a given power output (PO) can lead to stabilization of P_i_ at a steady state less than Pi_peak_ and continuation of exercise potentially ad infinitum, while in untrained muscle, P_i_ reaches Pi_peak_ after a certain time, and exercise terminates because of fatigue. Therefore, PO that was greater than critical power (CP) before training is less than CP after training. In other words, the system transitions from the very heavy exercise domain to the heavy exercise domain. Finally, the decreased rise in P_i_ (and changes in other metabolites) in moderate exercise of a given power output (or in primary phase II of the P_i_ on-kinetics in exercises of higher intensity) leads to faster reaching by P_i_ and ADP (both stimulate OXPHOS during rest-to-work transition) of the working steady state, faster reaching by $\overset{\cdot }{V}$O_2_ of the active steady state and thus shortening of the $\overset{\cdot }{V}$O_2_ on-kinetics transition time: t_0.63_. 

A possible training-induced decrease in Pi_peak_ diminishes the effect of the elevated OXPHOS activity on $\overset{\cdot }{V}$O_2max_ and CP (but not t_0.63_). At a lowered Pi_peak_, P_i_ reaches Pi_peak_ faster, $\overset{\cdot }{V}$O_2_ has less time to increase, and thus, the exercise duration is shortened, and $\overset{\cdot }{V}$O_2max_ falls. The steady-state P_i_ value for a given PO at an unchanged Pi_peak_ can become greater than Pi_peak_ at a diminished Pi_peak_; as a consequence, P_i_ would reach Pi_peak_ instead of stabilizing at a steady state less than Pi_peak_, exercise would be terminated because of fatigue instead of continuing potentially ad infinitum, CP would become less than PO, and the system would pass from the heavy exercise-intensity domain to very heavy exercise-intensity domain.

## Data Availability

Not applicable.
